# Distance Measurement and Error Compensation of High-Speed Coaxial Rotor Blades Based on Coded Ultrasonic Ranging

**DOI:** 10.3390/mi16010061

**Published:** 2024-12-31

**Authors:** Yaohuan Lu, Shan Zhang, Wenchuan Hu, Zhen Qiu, Zurong Qiu, Yongqiang Qiu

**Affiliations:** 1 State Key Laboratory of Precision Measuring Technology and Instruments, Tianjin University, Tianjin 300072, China; luyaohuan@tju.edu.cn (Y.L.); fami_z34@tju.edu.cn (S.Z.); 2 College of Mechanical Engineering, Tianjin University of Technology and Education, Tianjin 300355, China; huwenchuan@tute.edu.cn; 3 School of Engineering, University of Bolton, Bolton BL3 5AB, UK; z.qiu@bolton.ac.uk; 4 School of Engineering, Liverpool John Moores University, Liverpool L3 3AF, UK

**Keywords:** blade tip distance, coded ultrasonic ranging, dynamic measurement, error compensation

## Abstract

Coaxial rotor helicopters have many advantages and have a wide range of civilian and military applications; however, there is a risk of blade collision between the upper and lower rotor blades, and the challenge still exists in balancing rotor parameters and flight control. In this paper, a blade tip distance measurement method based on coded ultrasonic ranging and phase triggering is proposed to tackle this measurement environment and expand the application of ultrasonic ranging in high-speed dynamic measurement. The time of flight (*Tof*) of coded ultrasonic ranging is calculated by the amplitude threshold improvement method and cross-correlation method, and the sound velocity is compensated by a proposed multi-factor compensation method. The static distance error of coded ranging with different codes are all within ±0.5 mm in the range of 10–1000 mm. The measurement error characteristics under different trigger phases and different rotational speeds are studied, and the error model is fitted by the back-propagation neural network method. After compensation, the vertical distance measurement errors are within ±2 mm in the range of 100–1000 mm under the condition that the rotational speed of the blade is up to 1020 RPM. It also provides a potential solution for other high-speed measurement problems.

## 1. Introduction

Compared with the single-rotor helicopter, the coaxial rotor helicopter not only has the advantages of smaller size, good stability, and increased lift and efficiency but also has better handling performance, climb rate, hover ceiling, and flight range, and eliminates the hidden danger of tail rotor failure so that it has a higher survival rate on the battlefield [1,2,3,4]. However, this coaxial design presents a hazard, which is the motion interference between the upper and lower blades when flying in a complex environment. In some cases, the upper and lower blades may collide. One solution is to reserve a large enough space between the upper rotor and lower rotor, but it will reduce lift power and lower maneuver performance. Therefore, if the distance between the blade tips can be measured in real time, then the space between the upper rotor and lower rotor can be optimized to ensure the performance of the helicopter while avoiding blade collision. Flight parameters can also be adjusted in time according to the changing trend of the distance to ensure flight safety under special circumstances.

The measurement methods that have been or may be applied to the blade tip distance include indirect measurement and direct measurement.

A typical indirect measurement method is to use fiber optic sensors arranged on the surface of the blade and then to obtain the upper and lower blade tip positions from the blade deflection curve [5,6]. However, the decoupling of blade deformation is difficult, and a larger error will occur in calculating the blade tip distance due to the cumulative effect of errors at the tip position.

Direct measurement methods are based on distance measurement sensors. Laser, vision/camera, electromagnetic waves, and ultrasound are common means of distance measurement.

Laser rangefinders can achieve high precision with a high measurement speed [7]. However, it is not suitable for dynamic measurement due to the directivity of the laser beam. If it is installed on the blade tip, the attitude change of the blade makes it impossible to ensure that the measurement points are at the targeted position during blade intersections.

Visual measurement is a common method to measure blade tip displacement, including monocular vision and multi-camera vision (binocular vision and trinocular vision) [8,9,10,11,12,13]. A single camera can be installed on the helicopter fuselage due to its relatively small size; however, when measuring the blade tip distance, the upper and lower blades can block each other within the camera’s view, resulting in the blade tip distance not being measured at the intersection. When measuring with multiple cameras, the cameras are mounted on the ground, and the positions of the upper and lower blade tips are obtained. The use of multiple cameras can solve the view-blocking problem; however, due to the large size of the device, it is only suitable for ground measurement on landed helicopters.

Electromagnetic measurement has fast measurement speed and strong adaptability to the environment. The measurement method of blade tip distance based on electromagnetic waves was first proposed in [14] by embedding electric or magnetic field antennas in the blade tips. However, in practical applications, there is a problem of low resolution. To solve this problem, researchers [15] proposed a variety of algorithms to optimize signal processing to improve measurement accuracy, but it was only validated for static tests.

Ultrasonic measurement is similar to electromagnetic measurement. The difference lies in the types of waves used. Compared with electromagnetic waves, the propagation speed of ultrasound is slower and suitable for short-range measurements [16] but provides higher measurement resolution, and there is no hidden danger of electromagnetic interference. When ultrasonic distance measurement is adopted, the challenge lies in the contradiction between the high-speed rotating motion of the blade and the slow propagation speed of the ultrasonic wave in the air. Under normal circumstances, ultrasonic ranging can only carry out the wave of the next measurement after completing the signal reception of the previous measurement, while the propagation speed of ultrasonic is slow, resulting in slow measurement speed. Our previous research [17] proposed to solve the problem caused by the slow propagation of ultrasonic speed by triggering the transmitted signal in advance. However, the measurement signal only triggered once at each intersection may lead to signal loss when there are sufficient torsion movements or deformations of the blades. In addition, the maximum measurement error reaches 9.54 mm within 100–1000 mm.

In this paper, a measurement method based on coded ultrasonic ranging and phase triggering is proposed for blade tip distance measurement with improved measurement accuracy. Coded excitation is usually used to improve the signal-to-noise ratio of the signal and resolution or avoid crosstalk [18,19,20,21]. Here, coded excitation is first used in the ultrasonic ranging system to add different characteristics to different ranging signals so that multiple measurements can be achieved during a single intersection without waiting for the previous measurement to be completed and then transmitting the next measurement signal. This method provides a theoretical basis for the application of ultrasonic ranging in high-speed dynamic measurement. In addition, phase triggering ensures that the measurement can be completed during the intersection. In this way, it is easier to ensure the effective completion of the measurement during the intersection process and more suitable for high-speed measurement. However, the error of ultrasonic ranging is greatly affected by environmental factors, and it is applied to dynamic measurement in this paper, so the dynamic error characteristics are worth studying. The measurement under different trigger phases and different rotational speeds is studied, and the error compensation model is established to improve the dynamic measurement accuracy. The study of the error characteristics of dynamic measurement is of great significance for the application of ultrasonic ranging to the measurement of blade tip distance.

The paper is structured as follows: Section 2 introduces the calculation method of blade tip distance based on coded ultrasonic ranging and phase triggering and the blade tip distance measurement system; Section 3 presents the signal denoising and extraction method, the distance calculation method of coded ultrasonic ranging and error compensation for dynamic measurement; Section 4 presents the experimental results, including the results of static measurement and dynamic measurement; and Section 5 summarizes the advantages and disadvantages of the proposed method.

## 2. Measurement Method of Blade Tip Distance

### 2.1. Measurement Method Based on Coded Ultrasonic Ranging and Phase Triggering

**Figure 1:**
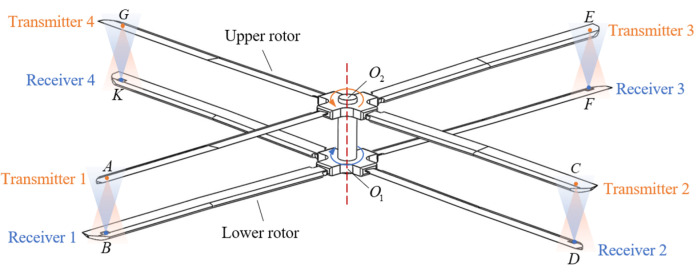
A schematic diagram of blade tip distance measurement scheme based on ultrasonic ranging.

Taking an eight-blade coaxial rotor helicopter as an example, Figure 1 shows the structure of the coaxial rotors: the upper rotor rotates counterclockwise, and the lower rotor rotates clockwise at the same rotation speed. Ultrasonic ranging includes two kinds: reflection mode and pitch-catch mode. The reflection mode uses the same transducer to transmit and receive ultrasonic waves, while the pitch-catch mode uses two transducers to transmit and receive ultrasonic waves, respectively. The reflection mode takes the whole reflecting surface as the measured object, resulting in its specific measured point being uncertain, while the pitch-catch mode can fix the position of the measured point, that is, the position of the receiver. The ultrasonic waves in reflection mode propagate twice as far as those in pitch-catch, resulting in the reduction in the signal-to-noise ratio of the received signal. In addition, due to the transmission of multiple ultrasonic measurement signals, the use of reflection mode may cause the transmitted signal and the received signal aliasing, which is not conducive to signal processing. Therefore, the pitch-catch mode is adopted. The transmitters facing downward are individually installed near the tip of the upper rotor blades, and the receivers facing upward are installed near the tip of the lower rotor blades. The upper and lower blades intersect 8 times per rotation, and the corresponding 4 tip distance values need to be measured at each intersection. The distance between the pair is calculated by the time of flight and the speed of the sound.

**Figure 2:**
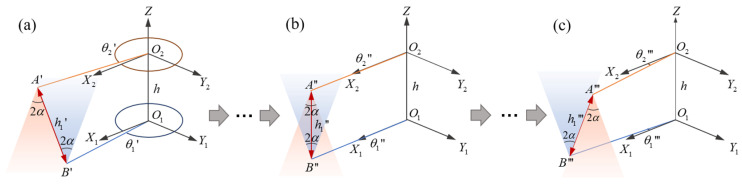
Blade tip distance measurement process at blade intersection: the (**a**) beginning, (**b**) middle and (**c**) end of blade intersection.

Ultrasonic propagation is directional [22,23], and the measurable range of the intersection process is defined as the 3D space area in which the receiver can receive the ultrasonic signals transmitted by the transmitter. Figure 2 shows the measurement process of blade tip distance at a certain intersection of blade tip *A* and blade tip *B*. The coordinate system is established with the upper and lower blades in a state of intersection, as shown in Figure 1. *O*_1_*Z* and *O*_2_*Z* are the axial direction of the hub, *O*_1_*O*_2_ = *h*, *O*_2_*A* = *O*_2_*C* = *O*_2_*E* = *O*_2_*G* = *O*_1_*B* = *O*_1_*D* = *O*_1_*F* = *O*_1_*K* = *r*, *O*_1_*X*_1_, *O*_1_*Y*_1_, *O*_2_*X*_2,_ and *O*_2_*Y*_2_ coincide with the directions of *O*_1_*B*, *O*_1_*D*, *O*_2_*A,* and *O*_2_*C*, respectively. If the rotation speed of the upper and lower rotors is ω, then the phase of blade *A* at time *t* is $\theta_{2}$, $\theta_{2}=(\omega t)\%(360)$ ($\theta_{2}\in [0{}^{\circ},360{}^{\circ})$), and the phase of blade *B* at time *t* is $\theta_{1}$, $\theta_{1}=-\theta_{2}$ ($\theta_{1}\in (-360{}^{\circ},0{}^{\circ}]$). In addition, the beam angle of the transmitter and receiver is 2α. Therefore, when the phase of the blade A′ is $\theta_{2}\prime =(360{}^{\circ}-(180{}^{\circ}\cdot h_{1}\cdot \tan\alpha )/2\pi r)$ in Figure 2a, the receiver can begin to receive the effective signal to complete the distance measurement, and $h_{1}$ is the difference in position between blade tip *A* and blade tip *B* in the direction of the *Z* axis. Next, the phase of the upper and lower blades is closer, and in the state shown in Figure 2b, that is $\theta_{2}\prime \prime =\theta_{1}\prime \prime =0{}^{\circ}$, the vertical blade tip distance can be measured. Until the blade moves to the position shown in Figure 2c, $\theta_{2}\prime \prime \prime =(180{}^{\circ}\cdot h_{1}\cdot \tan\alpha )/2\pi r$, the effective signal can no longer be received if it continues to rotate, thus ending the measurement of this intersection. In practical application, the aim is to measure the vertical distance of the blade tip. If the vertical distance cannot be measured in a certain intersection process, the measured value needs to be compensated according to the phase of the blade.

**Figure 3:**
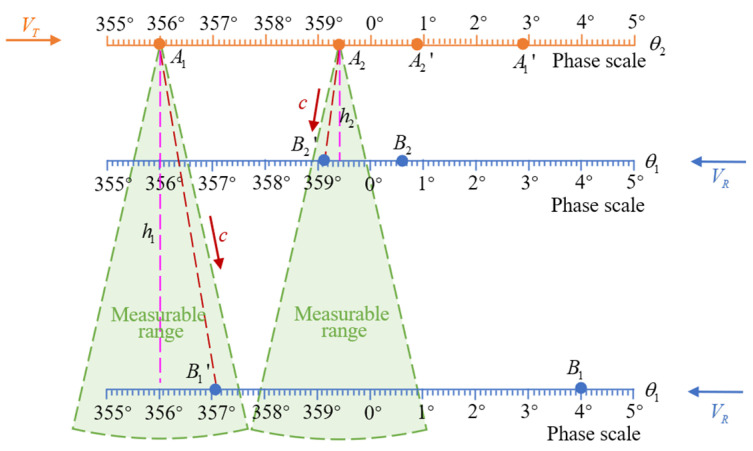
A schematic diagram of phase trigger measurement and vertical distance calculation.

The time of each blade intersection is very short since the blade tip moves fast, up to 200 m/s, and the propagation speed of ultrasound is relatively slow. To measure the effective distance during blade intersection, the method of coded ultrasonic ranging and phase triggering is proposed. Figure 3 illustrates the method in the form of a phase expansion diagram, and 2 transmitted signals at an intersection are taken as an example. The transmitter is set in advance to transmit a set of ultrasonic signals when the blade rotates to certain phases. The upper blade tip is at the position of *A*_1_ and *A*_2_ with $\theta_{2}=\beta_{1}$ and $\theta_{2}=\beta_{2}$ when transmitted signals. At the same time, the receiver is located at *B*_1_ and *B*_2_ with different vertical distances. And then, the transmitted ultrasonic signals propagate in the air, and the receiver rotates with the blade until the ultrasonic signal is received at the position of $B_{1}\prime$ and $B_{2}\prime$ within the measurable range. To obtain the vertical distance $h_{1}$ and $h_{2}$, the following formula is adopted.

(1) $$h_{i}=\sqrt{{\left(c\cdot \Delta t\right)}^{2}-{\left[2\cdot (360{}^{\circ}-\beta_{i})\cdot \frac{2\pi r}{360{}^{\circ}}-V_{R}\cdot \Delta t\right]}^{2}}$$

where $h_{i}$ is the vertical distance, *c* is the ultrasonic propagation speed, $V_{R}$ is the linear speed of the blade tip rotation, $\beta_{i}$ is the phase of the transmitter transmits the coded ultrasonic signals, and Δt is the time difference from the time of transmitting ultrasonic wave to the time of receiving ultrasonic wave.

If the measurement signal is only transmitted once at a fixed phase, then the distances of ultrasonic propagation of the transmitter are different at different vertical distances to be measured, so the phases of the signals received at the receiver are different, thus putting forward higher requirements for the measurable range of the transducer. If the measurement signals can be transmitted several times at the intersection, then the measurable range requirements of the transducer can be reduced, and the received signal energy is stronger since the phase of the receiver when the signal is received is closer to the phase of the transmitter. As shown in Figure 3, when $h_{1}$ and $h_{2}$ are measured, the transmitted signals used are signals of different trigger phases. Since the measured distance is unknown, it is necessary to trigger the measurement over a phase range. To achieve continuous measurement, the coded ranging method with 7-bit M-sequences is used within the measurement distance range of 100–1000 mm; that is, seven different measurement signals are transmitted. To make the two boundary values 1000 mm and 100 mm correspond to the trigger signal of the first code and the last code, respectively, it can be calculated as

(2) $$2\cdot (360{}^{\circ}-\beta )\cdot \frac{2\pi r}{360{}^{\circ}}/V_{R}=h_{1}/c$$

Two boundary phases can be obtained by substituting the two distance values corresponding to h1 into the above formula. Here, taking *r* = 3600 mm, *V_R_* = 170 m/s, and *c* = 340 m/s, the two boundary phases can be obtained as $\beta_{1}=355.8{}^{\circ}$ and $\beta_{7}=359.4{}^{\circ}$. Therefore, the trigger phases of the 7 coded ultrasonic signals are 355.8°, 356.4°, 357.0°, 357.6°, 358.2°, 358.8° and 359.4°. Continuous multiple measurements during each intersection prevent missing any signal that sometimes occurs in the single measurement method. The high signal-to-noise ratio is conducive to subsequent signal processing. In addition, when there are blade motions such as flapping, drag, and torsion, the continuous multiple measurement method is also more adaptable.

### 2.2. Measurement System

**Figure 4:**
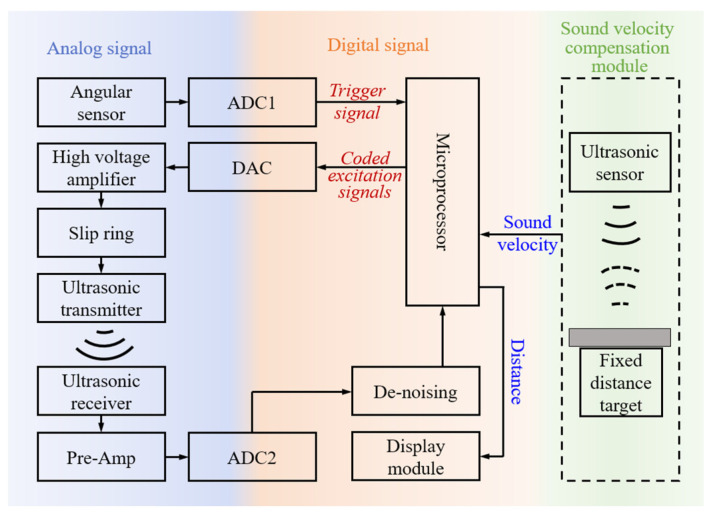
A block diagram of the blade tip distance measurement method based on coded ultrasonic ranging and phase triggering.

Figure 4 shows a block diagram of the proposed method. The measuring system is composed of an angle sensing module, an ultrasonic sensing module, and a signal processing module. The angle sensor is used to measure the blade rotation phase. After the microprocessor obtains the trigger signal based on the phase information, it transmits a series of coded excitation signals. The ultrasonic sensing module then starts to work, including the transmitter transmitting the excitation signal amplified by the high-voltage amplifier through the slip ring and the receiver receiving the ultrasonic signal propagating in the air and amplifying it to the signal processing module. The signal processing module is responsible for setting the phase of the transmitted signals, triggering the measurement at the fixed phase, and processing a set of coded signals received, including denoising, signal extraction, time of flight (*Tof*) calculation, etc. It is necessary to equip a sound velocity compensation module since the sound velocity is easily affected by environmental factors. To compensate for the sound velocity error caused by a variety of environmental factors, the method of ranging a fixed distance target with a calibrated ultrasonic sensor is adopted. Then, the distance between the transmitter and the receiver is calculated by the microprocessor based on the sound velocity obtained by the sound velocity compensation module and the *Tof* obtained by the microprocessor’s internal signal processing algorithm. This paper presents our research and results on the signal processing module with an emphasis on vertical distance calculation and dynamic measurement error analysis and compensation.

## 3. Signal Processing and Data Processing

### 3.1. Signal Denoising and Extraction

**Figure 5:**
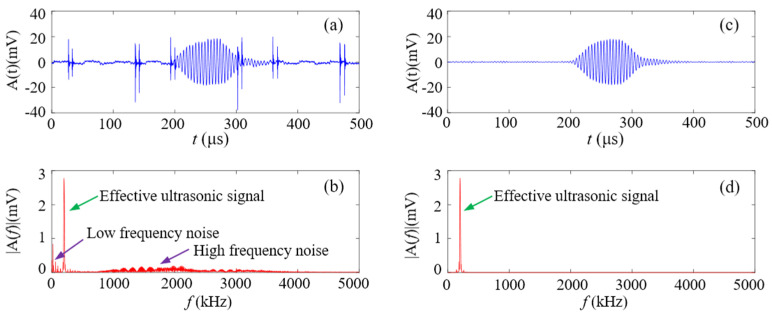
A received signal (**a**) before filtering in the time domain, (**b**) before filtering in the frequency domain, (**c**) after filtering in the time domain, and (**d**) after filtering in the frequency domain.

There are electronic noise and irrelevant clutter signals mixed with the received signals of the receiver, leading to a low signal-to-noise ratio (SNR), which affects ranging accuracy. As shown in Figure 5a,b, there are time domain and spectrum diagrams of the ultrasonic received signal mixed with noise at a measured distance of 500 mm. By analyzing the spectrum of the received signals, it is found that the received effective ultrasonic signals are near the center frequency of the transducer, 200 kHz, and the frequency band bandwidth is narrow, about 50 kHz, so the noise signal can be filtered by frequency domain filtering. A bandpass filter is a filter that passes through frequency components in a certain frequency range and attenuates frequency components in other ranges to low levels. It is suitable for denoising of ultrasonic signals. Since the center frequency of the transducer used is 200 kHz, the passband cutoff frequencies are set to *f_p_*_1_ = 150 kHz and *f_p_*_2_ = 250 kHz, the stopband cutoff frequencies are set to *f_s_*_1_ = 50 kHz and *f_s_*_2_ = 350 kHz, the maximum passband attenuation is 2 dB, and the minimum stopband attenuation is 30 dB. The time domain and spectrum of the filtered signal are shown in Figure 5c,d. It can be seen that both low- and high-frequency noise have been removed.

**Figure 6:**
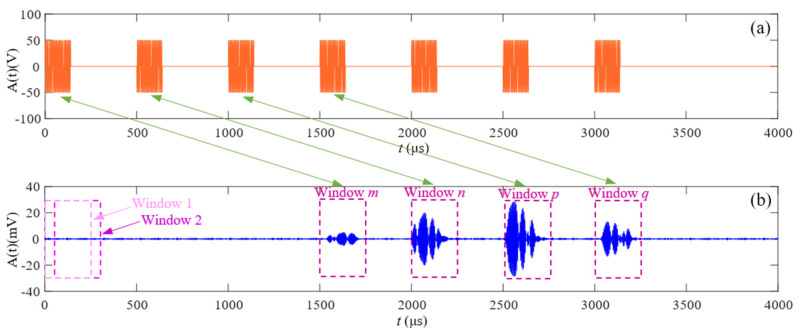
The (**a**) transmitted coded signals and (**b**) the received signals at an intersection.

To facilitate the subsequent analysis of the received signal, it is necessary to extract the effective measurement signal from the received signal for a fixed time length rather than processing the whole received signal. In addition, multiple measurement signals may be received due to a series of excitation codes in the continuous coded ultrasonic ranging in a single intersection. The received signals generated by different codes need to be labeled and saved separately. Figure 6a is the diagram of coded transmitted signals. After triggering the measurement, seven different coded signals are transmitted; Figure 6b is the diagram of the received signals. The signal can be received when the receiver is within the measurable range since the receiver is in motion, and four coded signals are received at this intersection. The steps of extracting the received signal based on the sliding window method [17] proposed in this paper are as follows:

(i) Definition of the window width *N* and sliding unit *step*. The window width is related to the receiving signal duration, and the sliding step determines the amount of computation. The larger the sliding unit, the smaller the computation, but at the same time needs to ensure that the received signal can be captured. Here, set *N* = 250 μs and *step* = 1/5 *N* = 50 μs;

(ii) Setting the start and end point of the window. Here, the start point is the time of the trigger signal, and set *t_start_* = 0. The endpoint is the *Tof* of the farthest measured distance and set to *t_end_* = 1000 mm/c;

(iii) Setting the threshold value *w_threshold_* of the energy of the window, which is determined by the lowest energy at the farthest measured distance *w_min_*, here set *w_threshold_* = 0.5 *w_min_*;

(iv) Calculating the energy in windows 1 to *end* = [*t_end_*/*step*] + 1;

(v) Recording the window number for those energies that are greater than the threshold *w_threshold_*. If there are continuous values in the recorded window number, only the window number with the highest energy density in these windows is recorded. As shown in Figure 6, the recorded window serial numbers are *m*, *n*, *p*, and *q*.

Since the envelopes of the received signals are different, so the received signals can be decoded respectively to find the corresponding transmitted signals, and then the *Tof* of the coded signal can be calculated.

### 3.2. Distance Calculation Method of Coded Ultrasonic Ranging

The distance (*d*) between the transmitter and the receiver is calculated by multiplying the sound velocity (*c*) and the measured *Tof* between the transmitted signal and received signal, as below:

(3) d=c⋅Tof

#### 3.2.1. Calculation of Tof

In this paper, the amplitude threshold improvement method (ATIM) and the cross-correlation method (CCM) are used to calculate the *Tof*.

(a) Amplitude threshold improvement method

**Figure 7:**
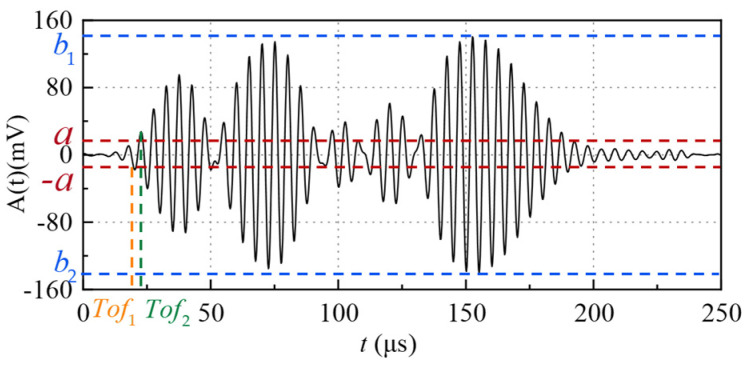
A schematic diagram of the amplitude threshold improvement method of the received signals at 100 mm.

The amplitude threshold method (ATM) [24] takes the first point where the received signal is greater than the threshold value as the signal starting point. The key to ATM lies in the selection of threshold value. Because the amplitude response of received signals with different codes is different, the selection of the threshold will also be different. The received signal of a code at 100 mm after signal extraction based on the sliding window method in Section 3.1 is taken as an example and shown in Figure 7. The recorded window serial number is *u*. The threshold *a* is selected according to the absolute value of the peak value *b* of the received signal amplitude, and *b* = max{|*b*_1_|,|*b*_2_|}, *a* = λ*b*, where λ is the threshold coefficient, which is in the interval [0.0625,0.1] and is related to the code. The threshold coefficient is determined by the experimental results of repeated experiments.

If only the amplitude threshold method is adopted, the moment when the absolute value of the first amplitude is greater than *a* is taken as the starting moment of the received signal, that is, the *Tof* is (u⋅step + *Tof*_1_). However, the initial moment of different measurements cannot be guaranteed to be in the same phase, so there will be errors. The form of the excitation signal affects the initial vibration direction of the received signal [25]. Therefore, the amplitude threshold improvement method is proposed. If the first period of the transmitted signal is in the form of −*sin*, the first point larger than the threshold and the phase is 90° is taken as the initial moment; on the contrary, if the first period of the transmitted signal is in the form of *sin*, the first point larger than the threshold and with a phase of −90° is taken as the initial moment. As shown in Figure 7, the first period of the transmitted signal corresponding to this received signal is in the form of −*sin*, so the *Tof*_2_ with the phase of 90° is used as the initial moment, then the *Tof* is (u⋅step + *Tof*_2_).

The system error of distance calculation is caused by the thickness of the shell on the surface of the transducer and the calculation error of the initial moment, which can be compensated. By measuring the *Tof* at different distances in the range of 100–1000 mm, then the system delay error ΔTof is obtained by using the least square fitting method.

(4) $$\Delta \mathrm{Tof}=\frac{\sum_{i=1}^{v}\left(d_{\mathrm{Ri}}-\bar{d_{R}}\right)\left(d_{\mathrm{mi}}-\bar{d_{m}}\right)}{\sum_{i=1}^{v}{\left(d_{\mathrm{Ri}}-\bar{d_{R}}\right)}^{2}}$$

where *v* is the number of measurements, *d_Ri_* is the reference value of each measurement, and *d_mi_* is the measured value of each measurement.

Therefore, the revised *Tof* of ATM and ATIM are $\mathrm{To}f_{\mathrm{ATM}}=u\cdot \mathrm{step}+\mathrm{To}f_{1}-\Delta \mathrm{Tof}$ and $\mathrm{To}f_{\mathrm{ATIM}}=u\cdot \mathrm{step}+\mathrm{To}f_{2}-\Delta \mathrm{Tof}$, respectively.

(b) Cross-correlation method

The correlation method calculates the cross-correlation function between the extracted received signal and the transmitted signal and will produce a peak at the time delay between them [26,27]. The formula is as follows:

(5) $$r(k)=\sum_{n=-N}^{n=N}R(n)\cdot E(n-k)$$

where *R*(*n*) is the amplitude of the extracted received signal, and *E*(*n*) is the amplitude of the corresponding transmitted signal. The length of the transmitted signal is 140 μs, which is different from the received signal, so the duration of the received signal is guaranteed to be the same as the received signal through the zero-filling operation.

Then, the value of *k* corresponding to the maximum value of *r*(*k*) is the time delay of *R*(*n*) and *E*(*n*) and is defined as *Tof*_3_, so the *Tof* is (u⋅step + *Tof*_3_).

The system delay error ΔTof′ is calculated using the same method as above, and the revised *Tof* of CCM is $\mathrm{To}f_{\mathrm{CCM}}=u\cdot \mathrm{step}+\mathrm{To}f_{3}-\Delta \mathrm{Tof}\prime$.

#### 3.2.2. Sound Velocity Compensation Method

The sound velocity is easily affected by environmental factors, including temperature, humidity, pressure, etc. In general, the sound velocity compensation compensates for the influence of temperature due to its greatest influence. The temperature is measured in real time, and the sound velocity of the temperature compensation method is calculated by [28]

(6) $$c_{1}=331\sqrt{1+t_{C}/273.15}$$

To consider more environmental factors, a multi-factor compensation method is adopted, which involves using another calibrated ultrasonic sensor to measure a pre-calibrated distance *l* to calculate the real-time sound velocity, and the *Tof* is *Tof_calibration_*. This method works better than the method described in [17] because it only needs to measure once. The formula is as follows:

(7) $$c_{2}=l/\mathrm{To}f_{\mathrm{calibration}}$$

Therefore, the distances calculated by ATM, ATIM, and CCM with temperature compensation method are $d_{\mathrm{ATM1}}$, $d_{\mathrm{ATIM1}}$, and $d_{\mathrm{CCM1}}$, respectively, and $d_{\mathrm{ATM1}}=c_{1}\cdot \mathrm{To}f_{\mathrm{ATM}}$, $d_{\mathrm{ATIM1}}=c_{1}\cdot \mathrm{To}f_{\mathrm{ATIM}}$, and $d_{\mathrm{CCM1}}=c_{1}\cdot \mathrm{To}f_{\mathrm{CCM}}$. And the distances calculated by ATM, ATIM, and CCM with the multi-factor compensation method are $d_{\mathrm{ATM2}}$, $d_{\mathrm{ATIM2}}$, and $d_{\mathrm{CCM2}}$, respectively, and $d_{\mathrm{ATM2}}=c_{2}\cdot \mathrm{To}f_{\mathrm{ATM}}$, $d_{\mathrm{ATIM2}}=c_{2}\cdot \mathrm{To}f_{\mathrm{ATIM}}$, and $d_{\mathrm{CCM}2}=c_{2}\cdot \mathrm{To}f_{\mathrm{CCM}}$.

### 3.3. Error Compensation for Dynamic Measurements

In order to study the measurement when there is mutual motion between transmitter and receiver, the measurement state is analyzed as follows. As shown in Figure 2, when the distance is measured at each intersection, the measurement conditions may differ as follows: the phase of the transmitter transmitting the ultrasonic signal, the phase of the receiver receiving the ultrasonic signal, the velocity of the transmitter, the velocity of the receiver and the vertical distance being measured. Among these variables, the vertical distance is the measured value that needs to be output. Therefore, it is necessary to study the measurement when the transmitter and receiver are in different phases and at different velocities.

**Figure 8:**
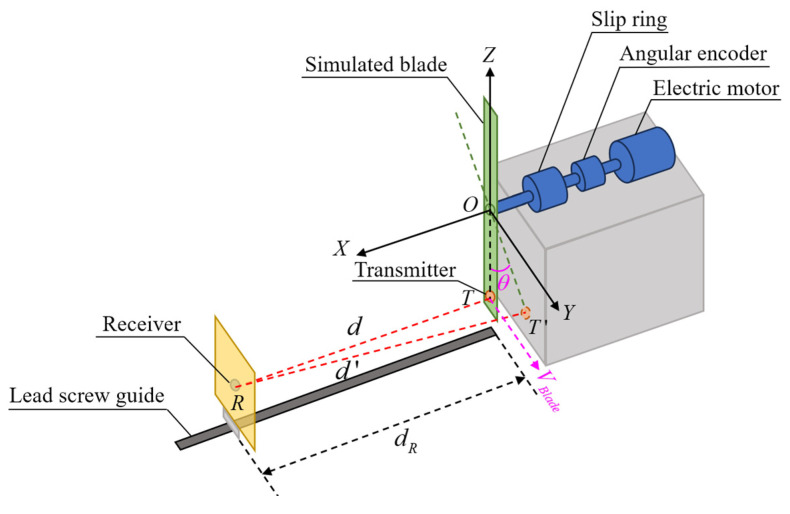
A schematic diagram of the setup of measurement experiments.

The schematic diagram of the setup of experiments is shown in Figure 8, which is mainly composed of an electric motor, an angular encoder, a slip ring, a simulated blade, and a lead screw guide. The horizontal structure is adopted to make the measuring device suitable for laboratory construction and ensure the premise of safety, and it can meet the requirements of measurement conditions at the same time. During the experiment, it is necessary to ensure that the distance reference value can be measured and controlled. The lead screw guide is used here, and the receiver is fixed on the guide. The speed of the transmitter is changed by changing the rotational speed of the simulated blade through the electric motor. The phase of the transmitter when transmitting the signal and the rotational speed of the simulated blade can be obtained by measuring the phase of the shafting through the angular encoder. Combined with the calibrated blade radius, the speed of the blade tip can be calculated. Therefore, the measurement device has the measurement conditions for different trigger phases and different blade tip speeds. With the simulated blade center as the coordinate origin, the vertical direction as *OZ*, the direction that is perpendicular to the blade rotation plane as *OX*, *OY* perpendicular to *OZ* and *OX*, *O*-*XYZ* coordinate system is established. The surface center points of the transmitter and receiver are T and R, and the coordinates in the *O-XYZ* coordinate system are (*x_T_*, *y_T_*, *z_T_*) and (*x_R_*, *y_R_*, *z_R_*), respectively. The speed of the transmitter is *V_Blade_*, and when it rotates to the position of T′, if the measurement is triggered at this time, its trigger phase is θ. θ is the angle of rotation of the blade rotates from *OT* to OT′. Here, we take θ∈(-180°,180°]. When the coordinate of the *Y*-axis of T′ is positive, θ is positive. On the contrary, when the coordinate of the *Y*-axis of T′ is negative, θ is negative. The vertical distance of the blade tip is the difference between the *X*-axis coordinates of the transmitter and the receiver, which is (*x_R_* − *x_T_*), so it is necessary to convert the distance measurement value *d* to vertical distance $d_{\mathrm{Vertical}}$ as follows.

(8) $$d_{\mathrm{Vertical}}=\sqrt{d^{2}-{\left(r-r\cdot \cos\theta \right)}^{2}-{\left(r\cdot \sin\theta \right)}^{2}}$$

where *r* is the distance between *O* and *T*.

The distance error Δd can be calculated by $\Delta d=d_{\mathrm{Vertical}}-d_{R}$, which is also studied with different θ and different *V_Blade_* in this research. The rotational speed of the coaxial helicopter is 400–500 revolutions per minute (RPM). Since this device is a single rotor rotation, the maximum rotational speed of the simulated blade needs to reach 1000 RPM.

**Figure 9:**
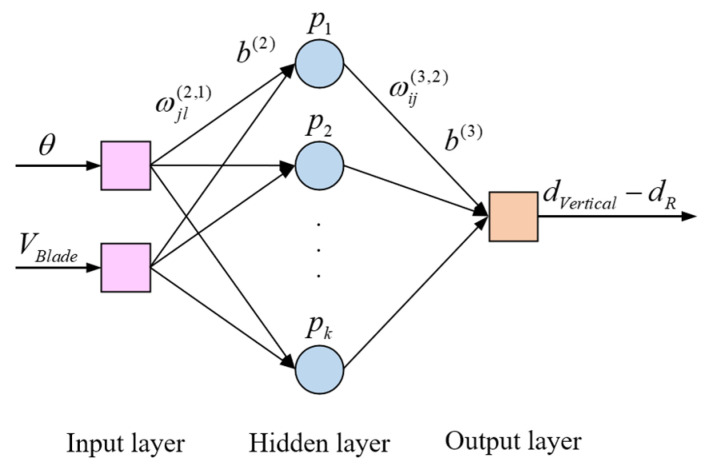
Error compensation model based on BP neural network.

Error compensation is a necessary process to improve measurement precision. It is necessary to analyze the factors that affect the accuracy of the measurement and perform data fitting for the errors of discrete distance points in the full range. The error model can be built by function fitting, but the error characteristics need to be known. In contrast, the back-propagation (BP) neural network algorithm [29,30] allows error characteristics not to be focused on, making it well suited for adoption in the error compensation process of dynamic distance measurement. BP neural network is a kind of artificial neural network based on the back-propagation algorithm, which is also one of the most widely used neural networks. BP neural network is composed of the input layer, hidden layer, and output layer, and the hidden layer can contain multiple neurons for processing the nonlinear mapping relationship of input data, as shown in Figure 9. So, it can effectively simulate the relationship between the trigger phase, the speed of the transmitter, and the corresponding error. The input layer contains two nodes, which are the trigger phase θ and the speed of the transmitter *V_Blade_*. The number of element nodes in the hidden layer is *k*, which is determined by the comparative experiment of different hidden layers. The output layer contains one node denoted by $d_{\mathrm{Vertical}}-d_{R}$, which is the output distance error. The connection weight and threshold from input layer to hidden layer are $\omega_{\mathrm{jl}}^{\left(2,1\right)}$ and $b^{\left(2\right)}$, and that from hidden layer to output layer are $\omega_{\mathrm{ij}}^{\left(3,2\right)}$ and $b^{\left(3\right)}$. The transfer functions of the hidden layer and the output layer are the *Tansig* function and the purlin function, respectively [31,32]. The *Tansig* function can effectively normalize the input data to another space, and the purlin function is a linear transfer function. The training function is the *Traingd* function, which is the batch gradient descent training function that can train a feedforward network with the fast BP algorithm.

The output of the hidden layer node is

(9) $$p_{j}=\rho \left(\omega_{j1}^{\left(2,1\right)}\cdot \theta +\omega_{j2}^{\left(2,1\right)}\cdot V_{\mathrm{Blade}}+b_{j}^{\left(2\right)}\right)$$

where ρ is the transfer function, $\omega_{j1}^{\left(2,1\right)}$ and $\omega_{j2}^{\left(2,1\right)}$ are the weights, and $b_{j}^{\left(2\right)}$ is the threshold of the hidden layer nerve unit.

The output layer node model is

(10) $$d_{\mathrm{Vertical}}-d_{R}=\sigma \left(\sum_{j=1}^{k}\omega_{\mathrm{ij}}^{\left(3,2\right)}\cdot p_{j}+b_{i}^{\left(3\right)}\right)$$

where σ is the transfer function, $\omega_{\mathrm{ij}}^{\left(3,2\right)}$ is the weight, and $b_{i}^{\left(3\right)}$ is the threshold of the output layer nerve unit.

By adjusting the connection weights and thresholds, the appropriate training model parameters are obtained. The training goal is to minimize the variance between the calculated value of the output layer and the actual value. Thus, the trained error model can be used to compensate for the error of the subsequent vertical distance measurement.

## 4. Results and Discussion

The experiments include both static and dynamic experiments. In the static experiments, both transmitter and receiver are at fixed distances without any rotation motion. The static experiments are used to test the distance calculation method and the multi-factor compensation method. The dynamic experiments, where the transmitter is rotating at certain speeds, are used to verify the feasibility of the vertical distance measurement of the blade tip based on ultrasonic ranging and the validity of the error compensation model.

### 4.1. Static Experiments

The blade tip distance measurement is based on coded ultrasonic ranging, and different codes may affect the ranging result. Therefore, the measurement under different coding excitation signals is studied in a static environment. In this paper, the measurement based on 7-bit M-sequences is studied and analyzed. A group of 7-bit M-sequences consists of seven sequences corresponding to seven different excitation signals [33]. Each excitation signal is composed of 7 code values. The code values of the M1 are M1-1[1,0,1,1,1,0,0], M1-2[0,1,1,1,0,0,1], M1-3[1,1,1,0,0,1,0], M1-4[1,1,0,0,1,0,1], M1-5[1,0,0,1,0,1,1], M1-6[0,0,1,0,1,1,1] and M1-7[0,1,0,1,1,1,0]. The code values of the M2 are M2-1[1,0,1,0,0,1,1], M2-2[0,1,0,0,1,1,1], M2-3[1,0,0,1,1,1,0], M2-4[0,0,1,1,1,0,1], M2-5[0,1,1,1,0,1,0], M2-6[1,1,1,0,1,0,0], and M2-7[1,1,0,1,0,0,1]. The numbers 1 and 0 are binary codes, and here, they correspond to the phase of the excitation signal. The number of periods of the excitation signal is determined by the measured distance range and the number of code values. When the code value is 1, the excitation signal is a 4-cycle sine with the frequency of the transducer center frequency, and conversely, when the excitation signal is 0, the excitation signal is a 4-cycle negative sine with a phase difference of 180 degrees of code value 1. The duration of each excitation signal is 28 transducer vibration cycles.

#### 4.1.1. Experiment Setup

**Figure 10:**
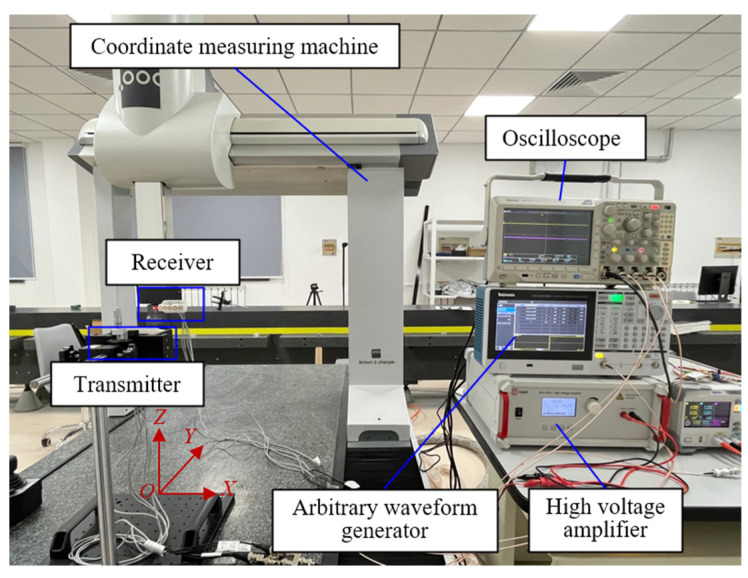
The experiment platform of ultrasonic static measurement.

The static experimental setup is shown in Figure 10. The measuring module is composed of an arbitrary waveform generator, a transmitter, a receiver, a high-voltage amplifier, and an oscilloscope and is based on the coordinate measuring machine (CMM). The coded excitation signals are generated by the arbitrary waveform generator, and the high-voltage amplifier amplifies the signal amplitude to 50 Vpp and then is transmitted by the transmitter. The receiver receives the propagated ultrasonic signal, which is collected by the oscilloscope. The transducer model is DYA-200-01B of Hangzhou Umbrella Automation Technology Co., Ltd., Hangzhou, China, the arbitrary waveform generator model is AFG31021 of Tektronix, Beaverton, OR, USA, the high-voltage amplifier model is ATA-2000 of Aigtek, Xi’an, China, the oscilloscope model is MDO3024 of Tektronix and the CCM model is Global Classic SR 07.10.07 of Hexagon Measurement Technology Ltd., Stockholm, Sweden. The center frequency of the transducer is 200 kHz, and the sampling rate of the oscilloscope is 5 MS/s. That is, 25 points are collected per cycle, which meets the Nyquist sampling principle. The reference distance between the transmitter and the receiver is provided by the CMM. The surface of the transmitter and receiver are aligned before the experiment. During the experiment, the transmitter is fixed, and the distance that the receiver moves with the measuring arm of the CMM is the reference value. The maximum permissible position error of the CCM is 2.3 µm. The measured distance is calculated according to the transmitted signal and the received signal. In this experiment, only the middle transducers in both transmitter and receiver are activated.

The measured distance is 100–1000 mm, where a measuring point is set every 100 mm. The different coded excitation signals are transmitted, and the received signals are collected at each measuring point. After all the coded ultrasonic ranging experiments of a single measuring point are completed, the measurement of the next measuring point is carried out to ensure the consistency of the measurement state.

#### 4.1.2. Results of Ranging

**Figure 11:**
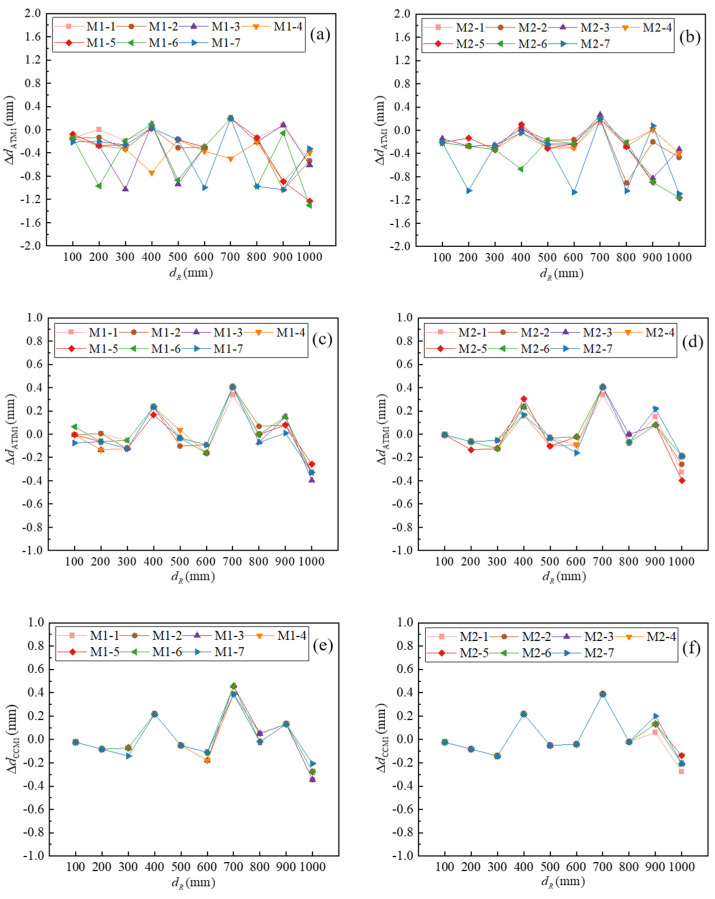
The errors of different methods: (**a**) ATM of code M1; (**b**) ATM of code M2; (**c**) ATIM of code M1; (**d**) ATIM of code M2; (**e**) CCM of code M1; (**f**) CCM of code M2.

For the two groups of code M1 and M2, the method in Section 3.2 is used to calculate the distance. According to the least square fitting compensation method of system delay error, ΔTof=22.45 μs and ΔTof′=33.50 μs can be obtained. According to the temperature compensation method and the multi-factor compensation method, $c_{1}=346.540\;m/s$ and $c_{1}=347.465\;m/s$ can be obtained. The reference distance is *d_R,_* which is obtained by the movement of the probe of the CMM. The errors of ATM, ATIM, and CCM with the multi-factor compensation method are $\Delta d_{\mathrm{ATM1}}$, $\Delta d_{\mathrm{ATIM1}}$, and $\Delta d_{\mathrm{CCM1}}$, respectively, and $\Delta d_{\mathrm{ATM1}}=d_{\mathrm{ATM1}}-d_{R}$, $\Delta d_{\mathrm{ATIM1}}=d_{\mathrm{ATIM1}}-d_{R}$, and $\Delta d_{\mathrm{CCM1}}=d_{\mathrm{CCM1}}-d_{R}$. The errors are shown in Figure 11.

As shown in Figure 11a,b, the error interval distributions of M1 and M2 with ATM are [−1.30 mm, 0.20 mm] and [−1.16 mm, 0.27 mm], respectively. The peak-to-peak value of the whole error range is 1.57 mm. Moreover, different codes have different measurement errors at the same reference distance. The difference between different codes when measuring the same distance can reach 1.11 mm, accounting for 71% of the peak-to-peak value, indicating that the difference in codes has a great impact on the measurement error when using ATM. Compared with ATM, the ATIM proposed in this paper is more adaptable to different coding conditions. As is shown in Figure 11c,d, the error interval distributions for both M1 and M2 with ATIM are [−0.40 mm, 0.41 mm], and the peak-to-peak value of the whole error range is 0.81 mm. The difference between different codes when measuring the same distance is 0.21 mm, accounting for 26% of the peak-to-peak value. It can be seen that the ATIM method improves the measurement accuracy, the error interval is reduced to 52% of ATM, and the influence of different codes on the measurement accuracy is reduced from 1.57 mm to 0.21 mm. As shown in Figure 11e,f, the error interval distributions of M1 and M2 with CCM are [−0.35 mm, 0.46 mm] and [−0.28 mm, 0.39 mm], respectively, the peak-to-peak value of the whole error range is 0.81 mm. The difference between different codes when measuring the same distance is 0.14 mm, accounting for 17% of the peak-to-peak value. CCM is also more accurate and less affected by coding than ATM. And the effect of coding on measurement is smaller than that of ATIM. On the other hand, ATIM and CCM have the same characteristics of the error curve, which can prove the reliability of the two methods. Therefore, ATM and CCM are suitable for distance calculation of coded ultrasonic ranging.

The comparison of measurement errors is shown in Table 1. The measurement error and relative error in Table 1 of this paper are calculated with M2 sequences using the CCM method. Compared with uncoded measurement results but using similar transducer center frequency [34], the measurement error in this paper is smaller, and the measurement range is larger. Compared with coded measurement results but using different transducer center frequencies, the measurement error and relative error in this paper are smaller, but the measurement range is also smaller. This is due to the influence of the transducer center frequency and the relatively small measurement range required by the blade tip distance.

Next, the experiment is repeated for the same distance, where 100 mm, 600 mm, and 1000 mm are selected to conduct 30 experiments, respectively, and the standard deviation is used to evaluate the stability of the coded range. The results are shown in Table 2. The measurement repeatability is good since the maximum ratio of standard deviation to the reference value of measured distance is 0.15%.

**Figure 12:**
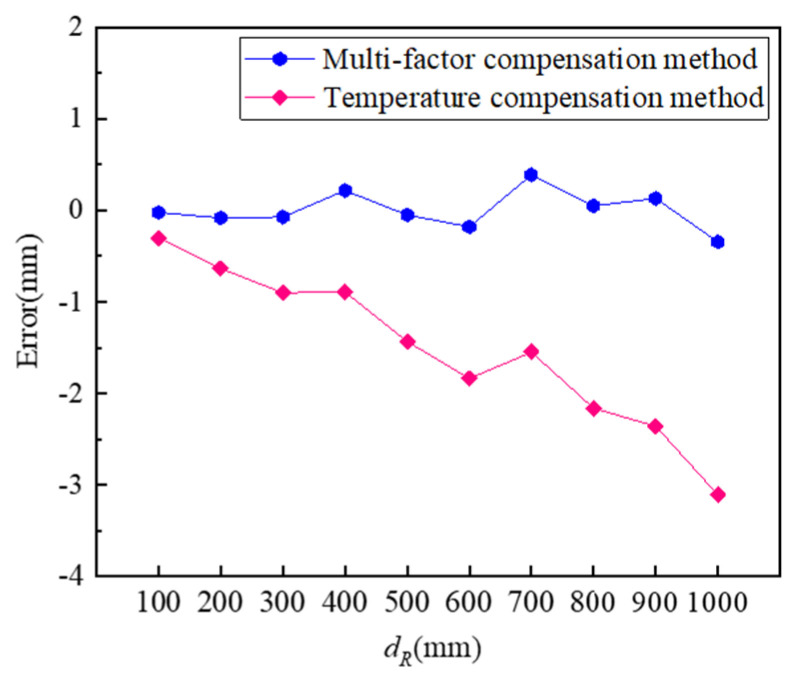
The comparison of the absolute errors for two different sound velocity compensation methods.

In order to verify the effect of the multi-factor compensation method, M1-1 with CCM was selected to be used in the experiment. The two sound velocity compensation methods obtain sound velocity values in the following ways: the temperature compensation method uses the data of the temperature sensor to calculate the sound velocity in real time according to Formula (6), and the multi-factor compensation method uses the calibrated ultrasonic sensor to measure a pre-calibrated distance 500 mm and then calculates the sound velocity according to Formula (7). The results are compared with the temperature compensation method, as shown in Figure 12. It shows that the multi-factor compensation method provides smaller errors. When the sound velocity error compensation effect is not good, the farther the measured distance is, the greater the distance measurement error will be. When the measured distance is 1000 mm, the error of the multi-factor compensation method is only 11% of the temperature compensation method, indicating that the multi-factor compensation method can compensate for the distance error more effectively.

### 4.2. Dynamic Measurements

The purpose of dynamic measurement is to verify the feasibility of vertical distance measurement in the state of mutual motion between transmitter and receiver and to explore the characteristics of distance measurement error under different trigger phases and different speeds in the range of 100–1000 mm.

#### 4.2.1. Experiment Devices

**Figure 13:**
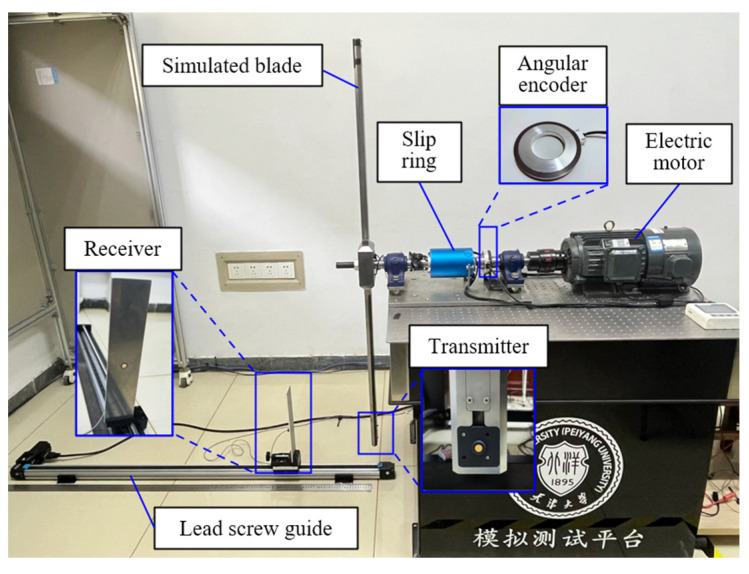
The experiment platform of ultrasonic dynamic measurement.

According to the research device shown in Figure 8, an ultrasonic dynamic measurement experimental setup that was used to validate the proposed blade tip distance measurement method is shown in Figure 13. The ultrasonic transmitter is embedded inside the blade tip of the simulated rotor, and its surface is consistent with the surface of the simulated blade. The receiver is fixed on the electric lead screw guide rail, and its surface is consistent with the corresponding mounting plate. The positioning precision of the lead screw guide is 0.03 mm, and the receiver can be moved on the guide rail. The transducer is the same as it is in the static test. Both the transmitter and receiver are the DYA-200-01B ultrasonic transducer. The excitation signal of the transmitter is a 200 kHz, 200 Vpp sine wave for 28 cycles, which is modulated by M-sequence, and here, M1 is selected. The 200 Vpp is used to ensure that the signal-to-noise ratio of the received signal from 1000 mm is sufficient under dynamic application. The excitation signal is transmitted through an arbitrary signal generator and amplified by a high-voltage amplifier, which then excites the transmitter through the slip ring. During the measurement process, the excitation signal is continuously transmitted with an interval of 0.9 ms, all the received signals are collected, and the effective received signals are extracted. The number of pulses of the induction head of the angular encoder is 2048 cycles per revolution. In order to study the measurement under different trigger phases and different speeds, a data acquisition card (multi-channel synchronous acquisition card with the maximum sampling rate of 10 MS/s, Chengdu MySoow Electric Co., Ltd., Chengdu, China) is used to collect the excitation signal of the transmitter, the received signal of the receiver and the phase signal of the angular encoder at the same time. In order to measure the vertical distance under different phases, the form of continuous transmission of measurement signals is taken here, and all effective received signals are collected. Since the phase signal and the excitation signal are collected at the same time, the phase of each transmitting signal can be calculated. The excitation signal and the received signal are used to calculate the *Tof,* and the phase signal is used to extract the phase θ and speed of the transmitter *V_Blade_* when it transmits the excitation signal.

Firstly, the aperture of the transmitter and the receiver are carefully aligned, and then the workbench of the electric lead screw guide is moved in the direction away from the transmitter. The moving distance of the workbench is taken as the reference distance. The measuring vertical distance range is 100–1000 mm, and one measuring point is selected every 100 mm. Limited by the performance of the electric motor, the rotational speed of the blade of the test platform is in the range of 300–1020 RPM. The electric motor speed was selected as 300 RPM, 550 RPM, 800 RPM, and 1020 RPM, respectively, for the experiment. Due to the low manual adjustment accuracy of the rotational speed, the actual speed of *V_Blade_* was calculated by the signal of the angular encoder combined with time information. In order to ensure that the state of the reference distance is consistent, the experiment under different speed conditions is completed at the same reference distance and then adjusted to the next measurement distance.

#### 4.2.2. Results of Ranging

**Figure 14:**
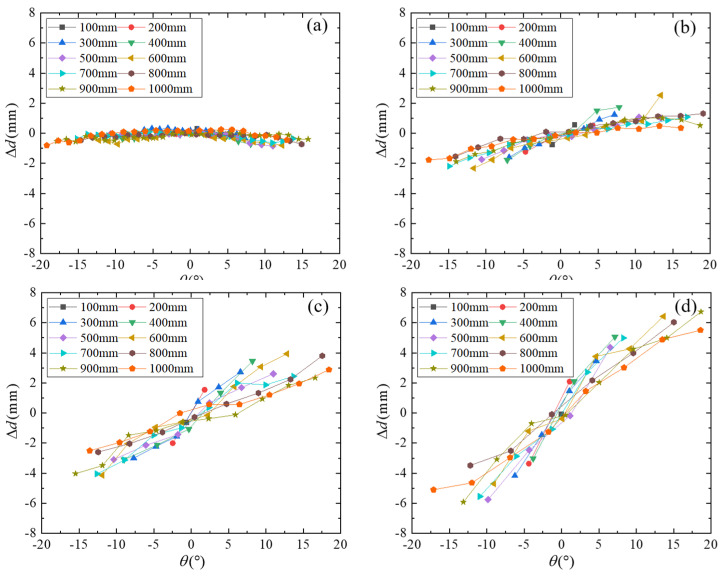
Distance errors under different trigger phases and different speeds (**a**) 300 RPM, (**b**) 550 RPM, (**c**) 800 RPM, and (**d**) 1020 RPM.

The distance errors under different trigger phases and different speeds in the range of 100–1000 mm are calculated, as shown in Figure 14. The actual speed *V_Blade_* range at the 300 RPM, 550 RPM, 800 RPM, and 1020 RPM rotational speed is 20.906–21.162 m/s, 40.438–40.947 m/s, 57.470–58.444 m/s, and 73.364–75.013 m/s, respectively. The distance error ranges of 300 RPM, 550 RPM, 800 RPM, and 1020 RPM are [−0.85 mm, 0.53 mm], [−2.33 mm, 2.52 mm], [−4.31 mm, 3.94 mm], and [−5.92 mm, 6.73 mm], respectively. With the increase in speed, the error range becomes larger, and the error is the smallest near zero phase. Most of the errors are positive in the positive phase, and on the contrary, most of the errors are negative in the negative phase, and the greater the speed, the more obvious the error characteristic. Analyzing the reason, when the phase is negative, the coordinate difference in the Y-axis direction when the transducer transmits the excitation signal is smaller and smaller, and on the contrary, when the phase is positive, the coordinate difference in the Y-axis direction is larger and larger, indicating that the coordinate position of the transmitted signal is a range, but only the starting point of the transmitted signal is calculated in the actual calculation. The above reasons cause the error trend of positive and negative phases to be opposite.

**Figure 15:**
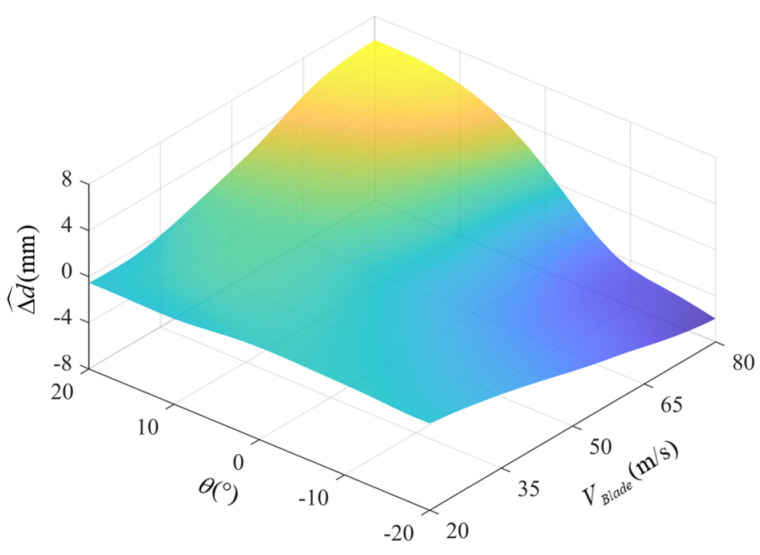
The error compensation model of dynamic measurement.

**Figure 16:**
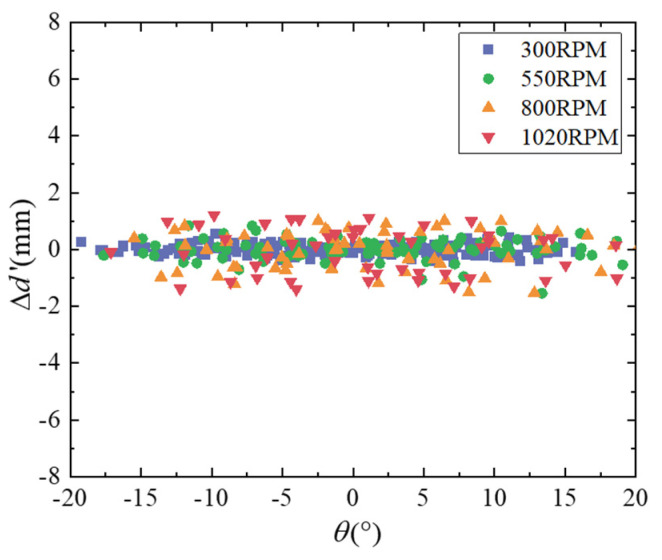
Distance errors under different trigger phases and different speeds after compensation.

The fitted error compensation model based on the BP neural network is shown in Figure 15, and the predicted error value is defined as $\hat{\Delta d}$. The compensation model is suitable for phase within ±20° and rotational speed between 300 RPM and 1020 RPM. Then, the error compensation model is used to compensate for the vertical distance measurement under trigger phases and different rotational speeds, and the vertical distance after compensation is $d_{\mathrm{Vertical}}\prime =d_{\mathrm{Vertical}}-\hat{\Delta d}$, so the distance error after compensation is $\Delta d\prime =d_{\mathrm{Vertical}}\prime -d_{R}$. The error results are shown in Figure 16, and the error distributions of 300 RPM, 550 RPM, 800 RPM, and 1020 RPM are [−0.44 mm, 0.53 mm], [−1.54 mm, 0.84 mm], [−1.53 mm, 1.01 mm], and [−1.40 mm, 1.21 mm], respectively.

Based on the statistical analysis, the evaluation of error compensation percentages represented by peak-to-peak value and standard deviation are shown in Table 3. The $Ratio_{\mathrm{peak}-\mathrm{to}-\mathrm{peak}\;\mathrm{value}}$ is the ratio of the peak-to-peak value of the error range after compensation to the error range before compensation for all distances at the same speed. The $Ratio_{\mathrm{standard}\;\mathrm{deviation}}$ is the ratio of the standard deviation of error results after compensation to the error results before compensation at all measurable phases and 1000 mm with the same speed of 3 revolutions. The smaller the value of $Ratio_{\mathrm{peak}-\mathrm{to}-\mathrm{peak}\;\mathrm{value}}$ and $Ratio_{\mathrm{standard}\;\mathrm{deviation}}$, the better the compensation effect.

As can be seen from Table 3, the error model can effectively compensate for the dynamic measurement error, and the higher the rotational speed, the better the compensation effect. The measurement error of vertical distance can reach within ±2.0 mm under the rotational speed of 300–1020 RPM.

## 5. Conclusions

This paper presents a method based on coded ultrasonic ranging and phase triggering for measuring the vertical blade tip distance of coaxial rotor helicopters. The feasibility of the method is proved theoretically and experimentally. Meanwhile, the signal processing method, the distance calculation method of coded ultrasonic ranging, and the dynamic measurement error compensation method are proposed.

The received signal is processed by bandpass filtering to denoise since the narrow bandwidth of the ultrasonic signal. The effective measurement signal is extracted for a fixed time length based on the sliding window method to fit the continuous measurement of coded ranging, and the different received signals are labeled and saved separately during signal extraction.

Static experiments have been used to verify the proposed method of *Tof* calculation based on ATIM and CCM, as well as the sound velocity compensation method based on the multi-factor compensation method. The distance measurement error can reach within ±0.5 mm in the range of 100–1000 mm. In addition, the influence of different codes on ranging accuracy has been verified at the same time. The ATIM and CCM are insensitive to codes and can be used for subsequent dynamic measurement.

Dynamic experiments have been used to verify the effectiveness of the proposed vertical blade tip distance measurement method based on ultrasonic ranging, and the measurement errors under different trigger phases and different speeds are studied. By studying the measurement error characteristics of different measurement conditions, an error compensation model based on the BP neural network algorithm is established. After error compensation, the error is reduced from [−5.92 mm, 6.73 mm] to [−1.54 mm, 1.21 mm], in the range of 100–1000 mm with the rotational speed of 300 RPM to 1020 RPM.

The comparison between the results of this paper and other references is as follows: [15] is a static measurement based on millimeter-wave radar with a maximum error of 5.7 mm, and [17] is a dynamic measurement based on ultrasound with a rotational speed of 400 RPM and the maximum error of 9.54 mm. In this paper, the measurement error is smaller, the maximum error is 2.0 mm, and the speed condition is more stringent, which can reach 1020 RPM. The effectiveness of the distance measurement method and the error compensation method proposed in this paper is illustrated. However, the measurement performance at speeds greater than 1020 RPM still needs to be further studied.

The method of measuring the distance of contra-rotating rotor blades based on ultrasonic ranging proposed in this paper provides a basis for other rotating measurements. The method of distance calculation and error compensation can also provide a reference for other similar measurements.

## Figures and Tables

**Table 1:** Comparison of the measurement errors.

	Measurement Error	Relative Error	Range	Transducer Center Frequency	Coded or Not
This paper	≤0.39 mm	≤0.06%	100–1000 mm	200 kHz	Coded
[34]	≤3.9 mm	/	30–450 mm	214 kHz	Not Coded
[35]	/	≤1%	400–2800 cm	55 kHz	Coded
[36]	≤29 mm	/	≤10 m	40 kHz	Coded

**Table 2:** Standard deviation of measurement results.

*d_R_*	100 mm	600 mm	1000 mm
σ	0.014 mm	0.091 mm	0.116 mm

**Table 3:** Evaluation of error compensation results of different speeds.

Rotational Speed/RPM	${\mathrm{Ratio}}_{\mathrm{peak}-\mathrm{to}-\mathrm{peak}\;\mathrm{value}}$	${\mathrm{Ratio}}_{\mathrm{standard}\;\mathrm{deviation}}$
300	69.7%	69.5%
550	49.1%	34.4%
800	31.5%	32.7%
1020	20.7%	19.5%

## Data Availability

Data are contained within the article.
